# Trypanosomosis by *Trypanosoma* (*Dutonella*) *vivax* in Ruminants in Brazil: Epidemiology, Clinical Signs, Pathology, Diagnosis, and Control

**DOI:** 10.3390/vetsci12090882

**Published:** 2025-09-12

**Authors:** Franklin Riet-Correa, Rodrigo Ferreira Krüger, Jose Augusto Bastos Afonso, João Alberto Negrão

**Affiliations:** 1 Postgraduate Program in Animal Science in the Tropics, Federal University of Bahia, Salvador 40170-110, Bahia, Brazil; 2 Ecology of Parasites and Vectors Group, Universidade Federal de Pelotas, Pelotas 96083-472, Rio Grande do Sul, Brazil; rodrigo.kruger@ufpel.edu.br; 3 Clínica de Bovinos, Universidade Federal Rural de Pernambuco—UFRPE, Garanhuns 52171-900, Pernambuco, Brazil; jose.basilva@ufrpe.br; 4 Basic Science Department, Faculty of Animal Science and Food Engineering (FZEA), University of São Paulo (USP), Av. Duque de Caxias Norte 225, Pirassununga 13635-900, São Paulo, Brazil; jnegrao@usp.br

**Keywords:** epidemiology, oxytocin administration, ruminants, *Stomoxys calcitrans*, Tabanidae, trypanosomosis, *Trypanosoma vivax*

## Abstract

In Brazil, *Trypanosoma vivax* was identified between 1970 and 1984 in buffalo, cattle, and sheep in northern Brazil. In the 1990s, the disease was diagnosed in the Pantanal of Mato Grosso. Currently, the disease is considered in enzootic stability in both regions. From 2006 to 2018, outbreaks of the disease associated with the presence of horseflies (Tabanidae) and stable flies (*Stomoxys calcitrans*) or transmitted by needle and syringe reuse were diagnosed in sheep, goats, and cattle during the rainy season in the semiarid region of Northeast Brazil, a region considered to have enzootic instability. Since 2013, numerous outbreaks have been diagnosed associated with oxytocin inoculation to induce milk letdown in dairy cows. Most outbreaks occur in cattle transported from areas of enzootic instability to areas of stability, or vice versa. Serological, parasitological, and molecular techniques have identified *T. vivax* in asymptomatic animals in all Brazilian regions. For disease prophylaxis, it is important not to reuse syringes and needles when administering medication and to avoid transporting animals during periods of insect abundance. It is also necessary to avoid the use of oxytocin or adopt safe injection practices, using disposable or disinfected needles and syringes only for cows with ineffective milk ejection.

## Simple Summary

In Brazil, trypanosomiasis caused by *Trypanosoma vivax* affects cattle, buffaloes, goats, and sheep. It is associated with horse flies (Tabanidae), stable flies (*Stomoxys calcitrans*), and the inappropriate reuse of needles, syringes, and medication vials in various animals. On dairy farms, the frequency of trypanosomosis has increased due to the daily administration of intravenous oxytocin to induce milk letdown. The main clinical signs of the disease include a decrease in milk production, progressive weight loss, hyperthermia, and anemia. Diagnosis should be made by identifying the parasite in the blood using parasitological or molecular methods. Once the disease is diagnosed, it is essential to interrupt its transmission and detect and treat affected animals. It is also essential to eliminate (in the case of injectable medications, including oxytocin) or minimize (in the case of insect vectors) transmission of the agent.

## 1. Introduction

Trypanosomosis caused by *Trypanosoma vivax* (Trypanosomatida, Trypanosomatidae) is a parasitic disease of ruminants and other species in Africa and South America. In Africa, the disease is transmitted by its biological vector, the tsetse fly [*Glossina* spp. (Diptera, Glossinidae)], and by mechanical vectors. In South America transmission occurs solely through mechanical vectors such as horse flies (Diptera, Tabanidae) and stable flies [*Stomoxys calcitrans* (Diptera, Muscidae)] [1,2], as well as through the reuse of needles and syringes during the administration of medications or vaccines [3,4].

Since 2013, trypanosomosis has caused significant losses in the Southeast region and some states in the Central–West and Northeast regions among dairy cattle treated daily with oxytocin to induce milk letdown. This practice has led to outbreaks with high morbidity and mortality [5,6,7,8,9,10,11,12,13], highlighting the need for effective control measures. This review aims to disseminate knowledge on the disease and establish strategies for its control.

The first diagnosis of trypanosomosis caused by *T. vivax* in South America was recorded in French Guiana in 1919. This parasite was likely introduced to South America from Africa around 1830, with the transport of infected cattle from Senegal [14,15]. In Brazil, *T. vivax* was identified between 1970 and 1984 in buffalo, cattle, and sheep in Northern Brazil [16,17,18,19]. In the 1990s, the disease was diagnosed in the Pantanal of Mato Grosso [19]. Currently, trypanosomosis and trypanosomiasis caused by *T. vivax* have been diagnosed in all South American countries except Uruguay and Chile.

Recently, in Brazil, the importance and economic impact of trypanosomosis have significantly increased due to the transmission of the disease through daily injections of oxytocin in lactating dairy cows [5,6,7,8,9,10,11,12,13,20,21,22,23,24]. In this manuscript, we review the infection by *T. vivax* in ruminants in Brazil, focusing mainly on aspects related to the epidemiology, clinical signs, pathology, diagnosis, and control of the disease. We searched PubMed, CAB Direct, and SciELO using the terms “*Trypanosoma vivax*”, “trypanosomosis”, “trypanosomiasis”, “cattle”, “bovine”, “goats”, “sheep”, “buffaloes”, “Brazil”, “South America”, and “Central America”, along with the corresponding words in Portuguese and Spanish; because many relevant articles are not indexed in those databases, we also searched Google Scholar with the same terms in English, Portuguese, and Spanish. The search covered the period from the first diagnosis of *T. vivax* trypanosomosis in cattle in South America (French Guiana in 1919) through 2025. In total, 412 papers were reviewed (including selected literature on babesiosis, anaplasmosis, and other hemoparasites for context), of which 106 met our inclusion criteria and were included in this review.

## 2. Epidemiology

In Brazil, trypanosomiasis was first reported in cattle in 1946, when the veterinarian José Lobato Boulhosa reported to the Animal Defense Office of the Ministry of Agriculture that he found trypanosomes in the blood of two cows in the Bragantina microregion of the state of Pará [19]. Later, *T. vivax* was identified at the Evandro Chagas Institute in a buffalo from a herd in Pará where the animals were losing weight [19]. Between 1969 and 1972, trypanosomosis was diagnosed on four farms in the state of Pará and one in the state of Amapá [16]. In at least one of these farms, the disease occurred after the introduction of animals from another region that had a high number of hematophagous insects. The disease was reproduced in sheep after inoculation of blood from animals with clinical signs, and the sick animals recovered after treatment with ethidium bromide [16]. In Amapá, during an outbreak of buffalo mortality, blood smears from 32 animals were examined, detecting *T. vivax* in 25% of them [17]. On the same farm, three years later, 8.9% of buffaloes and 7.6% of cattle were found to be infected with *T. vivax* [18]. Currently, due to the absence of outbreaks and the presence of animals with antibodies (Table 1) and asymptomatic carrier animals (Table 2), we consider the northern region as an area of enzootic stability.

In 1995, outbreaks of trypanosomosis caused by *T. vivax* were reported in the Pantanal of Mato Grosso in cattle that exhibited clinical signs such as fever, lethargy, loss of appetite, weakness, lacrimation, dysentery, abortion, and weight loss [25]. At that time, the authors suggested that the disease could lead to significant economic losses in the Brazilian Pantanal. However, no further outbreaks were recorded, and it was subsequently demonstrated that the disease is in enzootic stability in the region [33,34].

From 2002 to 2015, 10 outbreaks of trypanosomosis in cattle associated with insect transmission were reported, especially during periods of high abundance of horse flies (Tabanidae) and/or *S. calcitrans* (Table 1). Eight of these outbreaks occurred in the northeast region, most of which resulted from the introduction of animals, apparently asymptomatic carriers, into herds that did not have antibodies, during times of insect abundance. In one of these outbreaks, reported in the semiarid region of Paraíba, in a herd of the Swiss Brown breed, it was demonstrated that, before the outbreak, there were no cattle with antibodies, and the disease occurred after the introduction of asymptomatic animals carrying *T. vivax*. At the end of the outbreak, antibodies were present in the entire herd, and two years after the outbreak, only two of 85 animals still had detectable antibodies against *T. vivax*, demonstrating that the area is typical of enzootic instability [1]. Subsequently, outbreaks were reported in adult cattle [27], calves [28], sheep, and goats [3,35] in the same state, with some of them caused by the introduction of animals from the outbreak reported previously [1].

In an outbreak in the state of Maranhão, also in northeastern Brazil, morbidity was 8.8% and mortality 2.1%; however, at the end of the outbreak, the prevalence of antibodies was 82.5%, suggesting the occurrence of numerous subclinical infections [31]. The authors suggest the possible role of capybaras [*Hydrochoerus hydrochaeris* (Rodentia, Caviidae)] and other wild animals as carriers of the parasite [31].

Most of the outbreaks shown in Table 1 occurred after the introduction of asymptomatic carrier cattle into herds that, theoretically, did not have antibodies. These introductions happened during the rainy season, when Tabanidae [1,11,27,28] are abundant. Some of these outbreaks were caused by *S. calcitrans* [11,27]. However, an outbreak affected cattle from São Paulo after being transported to a farm in the state of Tocantins. The occurrence of this outbreak suggests that the cattle were transferred from an area of enzootic instability to an area of enzootic stability, where there was a high frequency of Tabanidae [26]. There have also been cases in which, after the outbreak, cattle were purchased to replace those that had died, and these recently introduced bovines became ill [6]. In other outbreaks, the introduced animals became ill, and members of the resident herd were also affected later [6]. This suggest that the resident animals had some degree of resistance but became ill when the infecting dose was increased, due to the presence of cattle in the acute phase. In addition, occasionally, after the disease was diagnosed, some farmers sold all of their animals, spreading the disease to other farms. In conclusion, the transport of animals from areas of enzootic stability to areas of instability, or vice versa, is one of the most important epidemiological factors contributing to the occurrence of outbreaks.

A different epidemiological condition occurred in São Paulo during an outbreak observed in the dry season, which was associated with a high population of *S. calcitrans*. In this case, fertilization of sugarcane plantations surrounding dairy farms with biosolids residues from sugar and ethanol plants provided adequate organic matter to attract *S. calcitrans* and promote its development [2].

In Brazil, an experimental study with confined cattle did not endorse the transmission of *T. vivax* by *S. calcitrans* [36]. However, Muita et al. [37] demonstrated the transmission of *T. vivax* to rabbits that died of trypanosomosis after being infected by *S. calcitrans*. These results suggest that, in Brazil, the transmission of *T. vivax* by *S. calcitrans* can only occur under very special epidemiological conditions, when the fly population is very high [2]. Large populations of *S. calcitrans* have been described in areas of sugarcane mills in Southeastern Brazil following mechanized harvesting and fertigation with vinasse [38]. Cadioli et al. [2] also noted that a large population of *H. irritans* may play an important role in the transmission of *T. vivax*. In Colombia, Zapata Salas et al. [39] found *T. vivax* in the proboscis and thorax–abdomen of *Haematobia irritans* (Diptera, Muscidae) and *S. calcitrans* suggesting that the first one, which represented 97.1% of the fly population, was the main potential vector. However, the detection of *T. vivax* DNA in both the proboscis and thorax–abdomen of *H. irritans*, along with several biological and ecological characteristics of this species cast doubt on its role as an effective mechanical vector. First, *H. irritans* is highly dependent on the host’s body temperature and tends to remain on a single individual for prolonged periods, limiting opportunities for inter-host transmission. Second, females leave the host only briefly to oviposit on fresh dung, typically returning to the same host, further restricting contact with multiple animals. Third, their small size and reduced mouthparts imply a lower capacity to carry and transmit blood-borne pathogens mechanically. Therefore, despite representing most of the fly population, *H. irritans* is unlikely to play a significant epidemiological role compared to larger more mobile species such as *S. calcitrans* or horse flies (Tabanidae), which have greater dispersal capacity and are known to feed on multiple hosts.

In an outbreak in sheep in the semiarid region, the use of injectable drugs was apparently the main form of transmission during anthelmintic treatment or administration of other drugs [3]. In addition, in an outbreak in cattle in Maranhão, the administration of several drugs by sharing the same needles between animals was suggested as an important factor in the transmission of the disease [31]. This route of transmission is likely more frequent than reported, since, mainly in dairy cattle, parenteral administration of several drugs (vaccines and anthelmintics, among others) is very frequent. However, this practice has not been mentioned in most outbreaks as an important form of transmission.

*Trypanosoma vivax* can be transmitted through subcutaneous, intramuscular, and intravenous routes by using the same syringe and needle previously used in an animal with acute *T. vivax* infection. Out of 20 cattle inoculated subcutaneously with saline solution contaminated with *T. vivax*, three became infected; of 20 inoculated intramuscularly, nine became ill; and out of 20 inoculated intravenously, 15 became infected. The parasite transmission was also effective when it was mixed with drugs, including antibiotics, analgesics, anti-inflammatories, antipyretics, antiparasitics, vitamin complexes, reproductive hormones, and vaccines [4].

Another form of transmission of *T. vivax*, experimentally proven, is the use of rectal palpation gloves contaminated with blood, used on more than one animal, which can infect two to five other cattle, depending on the blood contamination of the gloves [40].

Batista et al. [28] observed high *T. vivax* parasitemia in 1–3-day-old calves, suggesting transplacental transmission. However, no transplacental transmission of *T. vivax* was demonstrated in experimental infections, suggesting that additional research is needed to demonstrate transplacental transmission [41]. The presence of *T. vivax* DNA was demonstrated in semen of experimentally infected cattle, but the possibility of *T. vivax* being sexually transmitted in cattle was not proven [42].

Carvalho et al. [43] identified *T. vivax* in an anemic bovine with low body score condition at a veterinary hospital in Minas Gerais. Later, on the farm where this bovine originated, it was discovered that the prevalence of serologically positive animals had increased from 7.4% to 47% between January 2007 and February 2009, without any clinical cases [44]. These results suggest that since the parasite was introduced to the farm, there has been a continuous increase in prevalence without clinical cases, establishing a situation of enzootic stability without significant losses. Due to the low frequency of outbreaks transmitted mechanically by insects or through the use of the same needle and syringes to inject different drugs, except oxytocin, it is likely that this type of transmission causes subclinical cases, allowing the herds to remain in enzootic stability without clinical signs.

**Figure 1:**
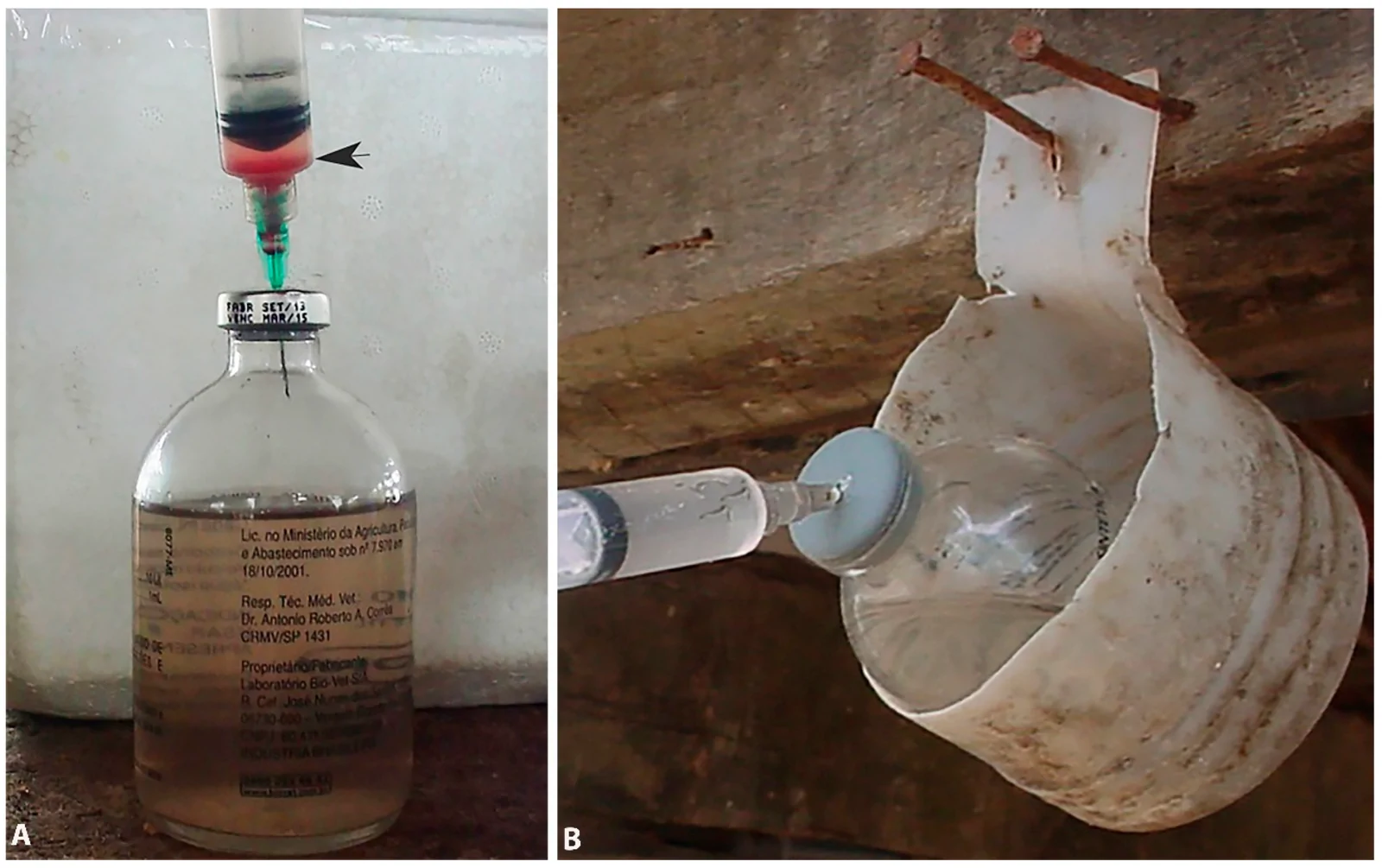
(**A**) Syringe being loaded into a vial of oxytocin. Note that there is a significant amount of blood in the syringe (arrow), coming from the previously treated cow. In addition, the content of the oxytocin vial is cloudy due to the presence of blood. (**B**) An example of how some farmers maintain the oxytocin that is being used in the milking parlor.

The first reported outbreak in Brazil associated with intravenous oxytocin administration for milk let down occurred in the state of Pernambuco in 2013 [11]. Subsequently, at least 38 outbreaks associated with oxytocin inoculation have been reported until 2025 (Table 2). These outbreaks occurred in the northeast, southeast, and Central–West regions, affecting lactating cows, mainly of the zebu breeds inoculated at each milking with oxytocin to induce milk letdown. Oxytocin is inoculated without adequate hygienic and sanitary conditions. The same needle, often contaminated with blood, is used to inoculate several animals, also contaminating the syringes and the vials containing oxytocin (Figure 1). Only in a few outbreaks has the disease affected dry cows or calves [9,11]; in these cases, the disease was apparently transmitted after the onset of the outbreak by the presence of hematophagous insects and/or probably by sharing syringes and needles for the inoculation of other drugs. In some outbreaks, a few lactating *Bos taurus* (Artiodactyla, Bovidae) cows (Holstein, Jersey or crossbred) were affected. In these cows, although not necessary, oxytocin was also used for milk letdown [5,9,23].

The morbidity and mortality rates observed in outbreaks caused by insect transmission (Table 1) are much lower than those caused by oxytocin inoculation (Table 2). These results suggest that when the parasite is transmitted by insect vectors, the morbidity and mortality are less severe compared to when the parasite is transmitted by daily injections of oxytocin. Outbreaks associated with oxytocin inoculation (Table 2) and, to a lesser extent, some insect-borne outbreaks (Table 1) have resulted in high morbidity and mortality rates. This is likely due to a lack of prior knowledge of the disease by veterinarians and diagnostic laboratories, leading to significant delays in diagnosis and therefore in the implementation of appropriate therapeutic and control measures.

Table 3 displays the frequency of antibodies in different regions of Brazil, while Table 4 shows the presence of *T. vivax* in herds that did not exhibit clinical signs of the disease (asymptomatic carriers). It is challenging to determine whether each region or state in Brazil is in enzootic stability or instability. However, the initial conclusion drawn is that the parasite is present in all regions of the country, with only two studies reporting no antibodies: one in the semiarid region of Paraíba [45] and another in the state of Paraná [46]. Despite the limited available data, we can define the situations in the following regions or states:

a. The outbreaks reported in Paraíba, Pernambuco, Ceará, and Rio Grande do Norte, along with the serological findings (Table 1 and Table 2) suggest that there is a region of enzootic instability in the Northeast, particularly in the semiarid region. In this area, prolonged droughts are common, and outbreaks tend to occur during the rainy season, coinciding with periods of increased Tabanidae abundance. This association persists despite the limited number of species identified in these states and the absence of studies addressing the seasonality of horseflies [47]. After an outbreak, the prevalence of animals with antibodies is high, but within 2 years the animals lose these antibodies, and apparently, the herd becomes susceptible again [1]. This state of enzootic instability in the semiarid region of the Northeast was also evidenced in a serological survey in the semiarid region of Paraíba, in which no animals with antibodies were found in 509 cows on 37 farms [45].
b. In contrast, the presence of antibodies, or even the parasite in low numbers, shows that in many regions the disease is in enzootic stability. This is evident in areas like the Pantanal of Mato Grosso and in the North region, where the parasite was identified or outbreaks occurred in the 1970s and 1990s [16,17,18,19]. Subsequently, the presence of antibodies or the parasite (Table 3 and Table 4) suggests that the disease remains in enzootic stability due to the high population of hematophagous insects in the region throughout the year.
c. Another region that appears to be well-defined is the South, encompassing the states of Rio Grande do Sul, Santa Catarina, and Paraná. Despite the presence of antibodies and parasites (Table 3 and Table 4), no outbreaks of the disease have been reported in this region. This is likely due to the temperate and subtropical climate, which only allows for seasonal activity of Tabanidae. Horse fly reproduction is limited to warmer periods and ceases during the cold seasons, preventing the growth of large vector populations for sustained transmission. In subtropical and temperate areas of southern South America such as Rio Grande do Sul and Uruguay, the absence of outbreaks can be partially explained by the consistently low population density of horse flies throughout the year and the ecological characteristics of the local biomes. Studies from these regions show that while vector activity occurs seasonally, the average number of horseflies captured per Malaise trap per week is significantly lower than in tropical regions [47,48,49,50]. For instance, in the Amazon Forest, the Adolpho Ducke Reserve reported averages exceeding 130 flies per Malaise trap per week [51], while the Colombian Amazon showed values close to 26 [52]. The annual average in the Pampa and Coastal Plain of Rio Grande do Sul was only 4.38 [49], and as low as 0.59 in livestock areas of Tacuarembó, Uruguay [50]. Even with intensive short-term sampling—such as a single summer week in the Coastal Plain of Rio Grande do Sul, southern Brazil using 98 Malaise traps, the peak observed was 37.57 flies per trap (RF Krüger, data not published), which is considered exceptional and not representative of typical long-term densities. This difference is closely related to the type of biome: tropical biomes like the Amazon and the Cerrado have environmental conditions favorable to the continuous reproduction of Tabanidae [51,53], while temperate ecosystems in the South, such as the Pampa and remnant Atlantic Forest areas, experience cold winters that limit Tabanidae activity and reproduction to a few warm months each year [49,50]. In contrast, horse fly populations in tropical climates remain abundant for longer periods and exhibit biannual peaks [54]. Therefore, even in regions where *T. vivax* circulation has been confirmed by serological or molecular methods, vector populations are likely insufficient to maintain effective mechanical transmission, explaining the absence of clinical outbreaks in these southern areas.
d. The Southeast region (states of Minas Gerais, Espírito Santo, São Paulo, and Rio de Janeiro) and parts of the Central-West (Goiás) and Northeast (Bahia, Alagoas, and Pernambuco, mainly in the Zona da Mata) are where the vast majority of outbreaks transmitted through the administration of oxytocin in zebu dairy cattle or their crosses have occurred. Trypanosomosis has acquired great economic importance in this region due to the losses it causes and all the necessary measures that must be taken to control it. In the Central–West region, in the Cerrado areas of Mato Grosso and Mato Grosso do Sul, the disease has not been observed; however, caution is still advised when introducing animals, especially on dairy farms that use oxytocin for milk release.

**Table 3:** Prevalence of antibodies against *Trypanosoma vivax* in cattle and buffaloes from different farms where clinical trypanosomosis did not occur.

State [Reference]	Species	Number		Technique	Prevalence
		Samples (*n*)	Farms/municipalities		
Mato Grosso do Sul [33]	Bovine	2508	Seven municipalities	ELISA	56%
Pará [33]	Bovine	1056	Five regions	ELISA	30.7%
Pará [55]	Bovine	246	NI ^a^	ELISA	93.1%
Mato Grosso do Sul [56]	Bovine	152	One	ELISA	48.5–52.6% ^a^
Minas Gerais [57]	Bovine	327	36	RIFI	16.2%
Pernambuco [58]	Bovine	2053	NI	IFA	13.93%
Alagoas [59]	Bovine	199	Four municipalities	RIFI	23.6%
Paraíba [47]	Bovine	509	37 farms	IFA	0%
Minas Gerais [60]	Bovine	2185	2185	RIFI	2.38%
Minas Gerais [48]	Bovine	400	40	RIFI	9.9%, 49.6% farms
Minas Gerais [61]	Bovine	101	Three	RIFI	63%
Parana [46]	Bovine	400	40 farms	IFA	0%
Mato Grosso do Sul-Pantanal [62]	Bovine	400	Five	ELISA	89.75%
Mato Grosso do Sul-Pantanal [63]	Bovine	200 200	NI	ELISA	98.5% adults 83.5% calves
Goias, São Paulo, Minas Gerais, Mato grosso [64]	Bovine	102	NI	ELISA	55%, 34.4%, 55%, 70%
Mato Grosso do Sul-Pantanal [65]	Bovine	170 312	Four Four	ELISA PCR-FFLB	50.59% 34.61%
Sta Catarina [66]	Bovine	146	Three	IFA	39%
Rio Grande do Sul [67]	Bovine	691	24	RIFI	24.6%
14 states [68]	Bovine	5114	NI	ELISA	56.5%
Maranhão [69]	Buffalo	116	Five municipalities	ELISA	79.31%
Santa Catarina [70]	Bovine	310	Six	RIFI	8%

^a^ Not informed.

**Table 4:** Microscopy or molecular identification of *Trypanosoma vivax* in farms from different Brazilian states where clinical trypanosomosis did not occur.

State [Reference]	Species	Number		Technique	Prevalence
		Samples	Farms		
Piaui [71]	Bovine	78	1	Microscopy	1.3%
Amapá [17,18]	Buffalo	125, 215	NI ^a^	Microscopy	25%, 8.9%
Amapá [18]	Bovine	210		Microscopy	7.6%
Pantanal-Mato Grosso do Sul and Paraguay [72]	Bovine Buffalo Sheep	355 43 83	9 2 2	PCR	44.7% 34.8% 37.3%
Mina Gerais [43]	Bovine	1	1	Microscopy-PCR	NI
Maranhão [73]	Bovine	31	1	Microscopy	3.2%
Rio Grande do Sul [74]	Bovine	1	1	Microscopy	NI
Maranhão [75]	Bovine	171 283	NI NI	PCR	6.21% 1.06%
Pará [76]	Buffalo	621 ^b^	60	PCR	1.89%
Pará [77]	Buffalo Bovine	89 61	3 2	FFLB	59.6% 44.3%
Minas Gerais [78]	Bovine	115	5	ELISA LAMP Woo	5.2% 4.3% 1.7%%
Santa Catarina [66]	Bovine	146	3	PCR	39%
Rio de Janeiro [79]	Bovine	389	15	PCR	11.6%

^a^ Not informed; ^b^ On these farms, *T. vivax* was also identified in 11 out of 184 ectoparasites [*Rhipicephalus* (*Boophilus*) *microplus* (Ixodida, Ixodidae), and *Haematopinus tuberculatus* (Phthiraptera, Haematopinidae)].

An important characteristic for the disease to remain in enzootic stability in South America is that in this continent the disease is transmitted mechanically, and the populations of *T. vivax* are genetically homogeneous; in contrast, in Africa, *T. vivax* presents greater antigenic diversity due to its transmission by its biological vector, the tsetse fly (*Glossina* spp.) [15,80,81,82]. However, the severity and frequency of the disease when transmitted by oxytocin inoculation suggest that outbreaks with greater lethality occur when the infecting dose is high, as in the case of oxytocin administration twice a day, especially when there are animals with high parasitemia in the herd. Although the criteria for enzootic stability are not clearly defined for trypanosomosis caused by *T. vivax* in South America, this criterion must vary, especially regarding the infective dose. It is likely that animals with certain antibody levels, who would not become ill when challenged by transmission by hematophagous insects, may become ill if the infective dose is very high, as in the case of intravenous inoculation of oxytocin twice daily.

The situation in regions with enzootic stability can be compared to a scenario of thick fever, caused by *Babesia* spp. (Piroplasmorida, Babesiidae) and *Anaplasma marginale* (Rickettsiales, Anaplasmataceae) in areas where *R. microplus* is present year-round, and cattle are constantly becoming infected and developing protective antibodies (concomitant immunity or premunition) [83]. This situation is defined as endemic (or enzootic) stability, which means a condition that implies a high incidence of organisms in cattle but rarely the presence of clinical disease [84]. However, herds with enzootic stability may still experience some subclinical losses or disease exacerbation despite being immune.

In the state of Paraíba, trypanosomosis was diagnosed in sheep and goats that were on the same farms as infected cattle during an outbreak. Approximately 25% of the sheep and goats showed clinical signs, and some died. After 6 months, the animals with clinical signs had recovered, and some had low parasitemia and normal temperature, demonstrating that they were asymptomatic carriers of the parasite. Therefore, after the outbreak, the flocks remained in enzootic stability, with no further clinical cases occurring [35]. In another outbreak in sheep, also in the semiarid region, the morbidity was 78.4%, and the mortality was 70.6%. It was suggested that *T. vivax* was introduced to the farm by buffalos that were asymptomatic carriers of the infection. In addition to the presence of Tabanidae that may have introduced *T. vivax* into the flock, it was suggested that the primary mode of transmission among sheep was through needles and syringes used for anthelmintic treatment and vaccination against clostridial diseases. The affected sheep were treated with oxytetracycline, sulfonamides, iron dextran, mineral supplements, vitamins, and amino acids [3].

## 3. Clinical Signs

After being infected with *T. vivax*, cattle may develop clinical signs depending on their nutritional condition or other intercurrent diseases. When the animals are in good health and nutrition, they respond well to the infection, do not develop clinical signs, and remain asymptomatic carriers [32,34,85]. It is also likely that the inoculation dose plays a crucial role in the development of the disease. This could explain why animals contaminated through daily oxytocin injections tend to experience more frequent and severe cases of the disease compared to transmission through insects or a single injection of other drugs.

**Figure 2:**
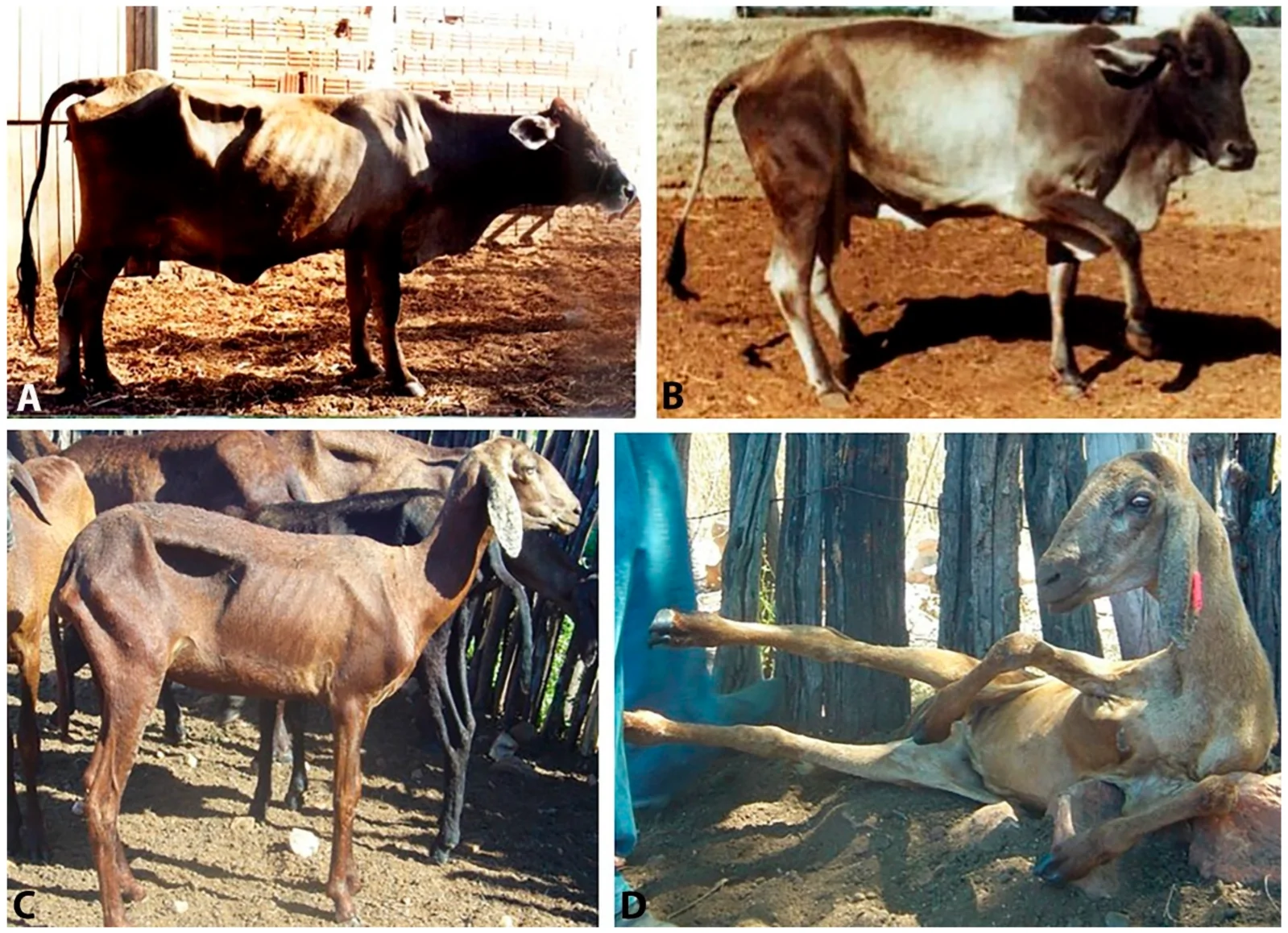
Cows and sheep showing signs of trypanosomosis. (**A**,**C**) Severe weight loss. (**B**) Hypermetria. (**D**) Convulsions.

Main clinical signs in cattle include a severe decrease in milk production, mild to moderate apathy, hyporexia, hyperthermia (40.5–42.0 °C), pale mucous membranes (with a hematocrit below 20%), and progressive substantial weight loss (Figure 2A) Diarrhea, submandibular edema, enlarged lymph nodes, mild jaundice, corneal opacity, sialorrhea, cough, and nasal discharge are also reported. Some animals show neurological signs, including incoordination, hypermetric gait (Figure 2B), muscle tremors, fasciculations, opisthotonos, nystagmus, blindness, strabismus, aimless wandering, aggressiveness, falls, convulsions, and paddling movements. Abortions, vaginal discharge, and the birth of weak calves that die after parturition are also reported. There is a severe (14% to 68%) decrease in milk production in the herd [1,2,6,7,8,9,13,21,27,31,85].

Death may occur 1–20 days after the onset of clinical signs. However, some animals, even without treatment, may progress to a chronic phase characterized by anemia and progressive weight loss. Animals that recover clinically do not regain milk production or the body condition they had prior to the disease [30,86]. Almost all animals with nervous signs die [1,2,7,9].

Batista et al. [30] observed significant differences in the reproductive parameters of 20 cows that had been naturally infected with *T. vivax* and had recovered from clinical signs and 20 uninfected cows. The group previously infected with *T. vivax* exhibited a significant increase in the following parameters: periods for the first postpartum estrus, calving intervals, estrus repetitions, first service period, and frequency of abortions. These effects are likely a consequence of weight loss and other clinical signs rather than the direct effect of the parasite on the reproductive system. Further studies are needed to confirm whether *T. vivax* is a primary cause of reproductive losses [20].

Anemia with a reduced globular volume, decreased number of red blood cells (hematocrit of 15–20% in most animals), and decreased hemoglobin concentration are the most common disorders in natural and experimental infections by *T. vivax* [1,2,6,21,27,85,87]. Leukogram values are inconsistent. Leukocytosis with lymphocytosis by neutrophilia and regenerative left shift or leukopenia may be observed, depending on the progression of the disease [2,6]. Plasma protein and fibrinogen values are also variable [2,6]. Enzymes indicative of liver, kidney, or muscle injuries do not show significant variations.

Clinical signs in sheep and goats are very similar to those in cattle: weakness and progressive weight loss (Figure 2C), anemia with pale mucous membranes, apathy, lack of appetite, enlarged lymph nodes, rough coat, submandibular edema, diarrhea, and recumbency [3,35]. Some animals may also present corneal opacity and blindness. Abortion or the birth of small and weak animals who die within three days of birth is also observed. In an outbreak in sheep, perinatal mortality, due to abortions and neonatal deaths, was almost 75% [3]. Some animals present nervous signs similar to those observed in cattle (Figure 2D) [3]. Many of the goats and sheep that are not treated recover spontaneously and become asymptomatic carriers, as do some animals who do not present clinical signs [3,35].

## 4. Pathology

Gross alterations are nonspecific and variable among necropsied animals. The main findings include the poor nutritional status, pale mucous membranes, presence of fluid in the cavities, serous atrophy of fat, moderately enlarged lymph nodes with whitish or hemorrhagic areas on the cut surface, enlarged spleen with rounded edges and prominence of the white pulp, enlarged liver, and ecchymoses or petechiae in the pericardium [1,9,27]. Mammary vein thrombosis and thromboembolic pneumonia have been reported in cows treated with oxytocin [6].

**Figure 3:**
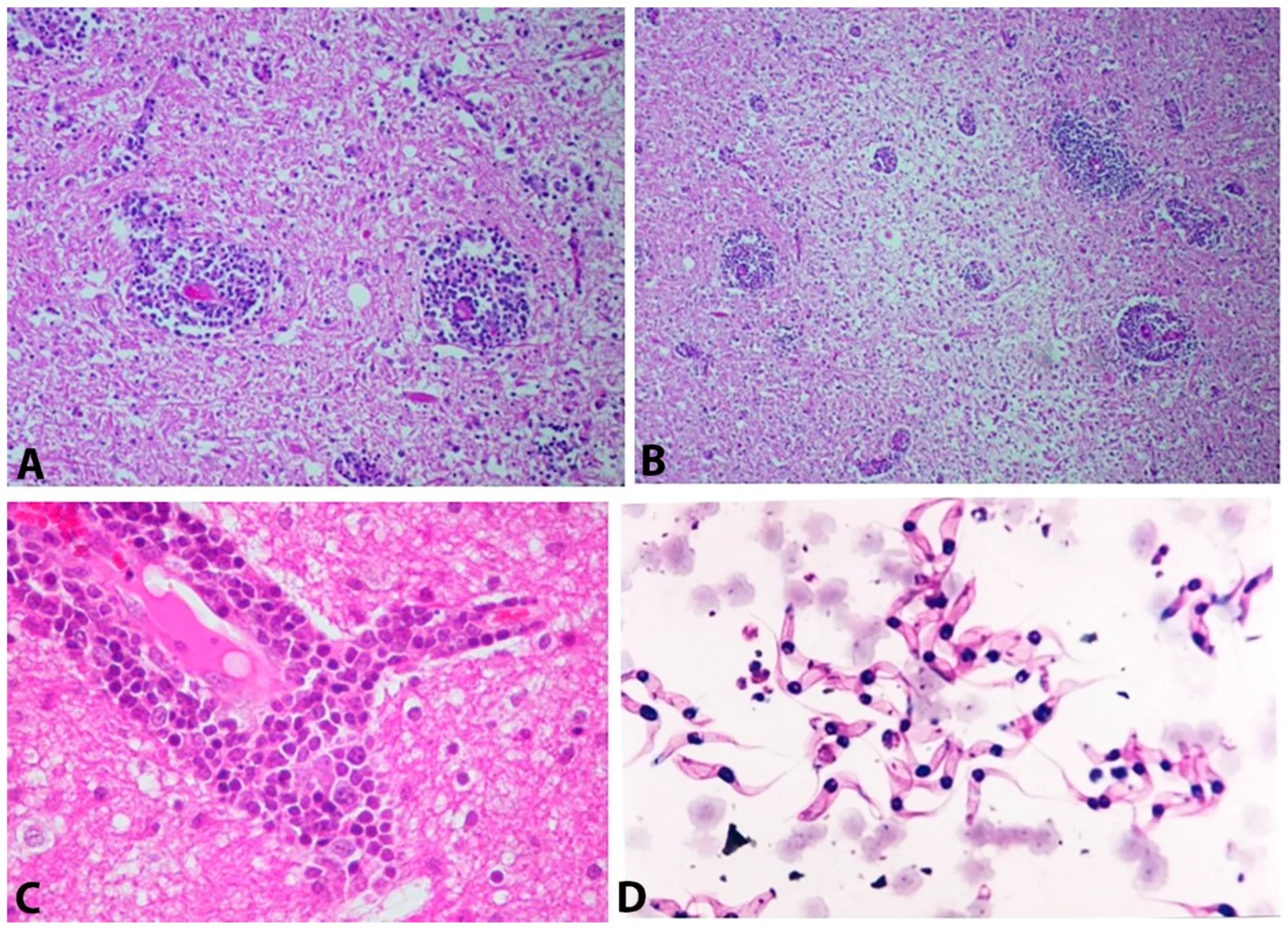
(**A**–**C**). Histology of the central nervous system of a cow (**A**,**B**) and a sheep (**C**) showing severe perivascular cuffing of mononuclear cells and gliosis. In the center of B, there is also a large clear area of rarefied white matter (Hematoxylyn and eosin stain). (**D**) Several trypomastigotes of *Trypanosoma vivax* from a microhematocrit tube stained by rapid panoptic method.

Histological lesions consist of multifocal lymphohistiocytic infiltrations in the myocardium, liver, kidney, adrenal glands and other organs, as well as follicular hyperplasia of lymph nodes. In the central nervous system, meningitis and myelitis are observed, with perivascular cuffing of mononuclear cells and macrophages, along with rarefied white matter and Gitter cells (Figure 3A,B). Plasma cells, eosinophils, multinucleated cells, and activated Mott cells are occasionally observed. Lesions are more severe in the brain than in the spinal cord, primarily affecting the white matter, meningeal vessels, and to a lesser extent in the gray matter [1,9]. Gross and histological lesions in sheep, including those of the nervous system (Figure 3C), are similar to those reported in cattle [3].

## 5. Diagnosis

A definitive diagnosis of trypanosomosis is essential for initiating treatment and controlling the disease. A presumptive diagnosis can be made based on clinical signs, necropsy lesions, and epidemiological data. However, it must be confirmed by identifying the parasite in the blood using parasitological or molecular methods. It is important to note that observing the parasite and/or detecting it through molecular methods does not confirm trypanosomiasis, as *T. vivax* can be found in asymptomatic carrier animals or animals with other diseases.

The main clinical signs to be considered are anemia and progressive rapid weight loss in a significant number of animals in the herd. A history of recent animal introductions, administration of medications with sharing of needles and syringes, and the presence of insect vectors (Tabanidae and *S. calcitrans*) are important pieces of information. Performing hemograms, especially hematocrit, is important to confirm anemia. Biochemical alterations did not show significant variations and are not helpful for diagnosis.

Given that animals showing nervous signs have 100% mortality rates, even with treatment, it is crucial to conduct necropsies on these animals. Histological studies of the nervous system and other organs can aid in the differential diagnosis. It is also important to necropsy animals that die without nervous signs.

Blood smears, stained using the rapid panoptic method, are effective for diagnosis in the early stages, when there is a high level of parasites in the blood. Another technique known as the Woo technique, involves concentrating blood samples in a microhematocrit tube. This method includes filling capillaries with blood, centrifuging the samples, and visualizing trypomastigotes (Figure 3D) that concentrate between the plasma and the buffy coat [6,8,88]. Additionally, needle aspiration of prescapular lymph nodes can be performed to observe *T. vivax* [88]. To enhance the efficiency of these techniques, which are always available, and generate a rapid diagnosis, it may be necessary to collect samples from several animals in the acute phase of the disease. Parasite morphometry or molecular methods should be utilized to distinguish *T. vivax* from other trypanosomes [43,44].

As the disease progresses, parasitemia becomes less frequent, and the intervals between parasitemia peaks increase, necessitating the use of other diagnostic methods. Molecular methods include conventional polymerase chain reaction (PCR) [88,89] and circular isothermal amplification of DNA (LAMP) [78,90]. Because molecular methods are more sensitive than parasitological methods, positive results are more likely to be obtained in asymptomatic carrier animals or those affected by another disease.

Serological methods should not be used to diagnose trypanosomosis caused by *T. vivax*. However, they are very useful for epidemiological studies, especially to determine whether a herd is in enzootic stability or not. The most frequently used techniques in serological diagnosis are the indirect immunofluorescence reaction (IFAT) and the enzyme-linked immunosorbent assay (ELISA) [88]. It should also be noted that in South America there may be cross reaction between *T. vivax* and two other trypanosomes infecting cattle—*T. evansi* and *T. theileri*—which have components in common with *T. vivax* and can lead to false positive results [82]. Furthermore, molecular detection methods such as PCR have shown good results, as they can determine the trypanosome species [5]. Reviews of the various parasitological, molecular, and serological methods for the diagnosis of trypanosomosis have been published elsewhere [88,89].

In the differential diagnosis of trypanosomosis caused by *T. vivax*, we preferentially include diseases that cause anemia and those that cause progressive weight loss. Among the diseases that cause anemia, we find several parasitic diseases, including babesiosis, anaplasmosis, hemonchosis, and fasciolosis. Babesiosis is more acute than trypanosomosis, presenting clinical signs such as hemoglobinuria, hyperthermia, and jaundice (more severe than occasionally described in trypanosomosis). In blood smears, numerous parasitized erythrocytes can be observed in the case of *Babesia bigemina*, but in the case of *Babesia bovis*, smears of capillary blood obtained by fingerstick, or smears of the cerebral cortex or other organs should be performed to observe *B. bovis* associated with the vascular endothelium. *Rhipicephalus* (*B.*) *microplus* is found in affected cattle, unless the herd has been treated. In the case of anaplasmosis, there is no hemoglobinuria, but anemia and jaundice are very marked, and numerous parasites can be found in the erythrocytes. Anaplasmosis, like trypanosomosis, can be transmitted by sharing needles or by hematophagous insects. Fascioliasis causes clinical signs that can be very similar to trypanosomosis, but the parasite is found in the liver during necropsies. Gastrointestinal parasitosis, especially hemonchosis, mainly affects young cattle up to 2 years of age. In the case of ostertagiosis, a rare disease in temperate climates, it can cause a disease very similar to trypanosomosis in cows over 2 years of age, with edema and weight loss. Diagnosis of gastrointestinal parasites is made through feces examination and/or necropsies.

Some plant poisonings that cause progressive weight loss must be differentiated from trypanosomosis. These include poisoning by *Solanum glaucophyllum* (Solanaceae) in the Pantanal of Mato Grosso and in the Taim region of Rio Grande do Sul and poisoning by *Pteridium* spp. (Dennstaedtiaceae) in several regions of Brazil [91]. Paratuberculosis, cobalt deficiency, a form of poisoning by *Brachiaria decumbens* (Poaceae) also associated with progressive weight loss, and eurythematosis by *Eurytrema coelomaticum* (Plagiorchiida, Dicrocoeliidae) [91,92] should also be included in the differential diagnosis. If neurological signs are observed, it is essential to differentiate trypanosomosis from other diseases such as rabies, hepatic encephalopathy, bovine herpesvirus-5 encephalitis, listeriosis, and several poisonings that present with nervous signs [91,92]. In trypanosomosis, it is important to confirm anemia and progressive weight loss in other animals in the herd that do not present nervous signs.

## 6. Control and Prophylaxis

Once the disease has been diagnosed, it is essential to stop its transmission. This requires detecting and treating the affected animals. Additionally, it is crucial to eliminate the transmission of the agent, such as through the discontinuation of injectable drugs like oxytocin or minimizing transmission through insect vectors.

Diminazen aceturate and isometamidium chloride are the two trypanocidal drugs licensed in Brazil, but there is evidence of resistance to these drugs [2,8,86]. Some authors recommend treating the entire herd with two strategic treatments spaced 120 days apart. However, to prevent resistance to trypanocidal in the Americas, the administration of these drugs should be restricted to affected animals only, and mass application in the herd should be avoided [27,93].

To prevent the spread of the disease within the herd, animals should be treated as soon as the first clinical signs are observed, such as a decrease in milk production, weight loss, fever, anorexia, and obtundation. This selective treatment allows animals with subclinical infections to develop concomitant immunity, leading to enzootic stability on the farm. Early identification of clinical cases can also be achieved by measuring the hematocrit and temperature of all animals. Early treatment when parasitemia is highest in the initial stages of the disease can quickly halt mechanical transmission by insects, if this is the primary mode of transmission [94].

Diminazene aceturate should be administered intramuscularly at doses ranging from 3.5 mg/kg to 8 mg/kg. It is advised to use the maximum dose to reduce the likelihood of resistance development. While this drug can be used prophylactically, its protection only lasts 2 to 4 weeks due to rapid elimination by the organism. Treatment with isometamidium chloride is also effective, administered intravenously or intramuscularly at a dose of 1.0 mg/kg [86]. It is important to note that stressed animals and those with concomitant diseases or poor nutritional status may relapse even after receiving specific treatment. After drug administration, milk from treated cows should be discarded for at least three days, and meat from treated animals should not be consumed for a minimum of 40 days, in accordance with the withdrawal periods for food safety.

To prevent the spread of *T. vivax*, vector control is essential. Strategies include the use of traps and insecticides [95]. For stable flies, environmental management practices such as the removal of soiled bedding, wet hay, and silage are critical to interrupt larval development. Biological control with parasitoid wasps, predatory beetles, and entomopathogenic organisms has shown variable success, depending on the environmental conditions [96]. Integrated Pest Management, combining chemical, biological, and mechanical strategies, is considered the most sustainable and effective approach [97].

In Brazil, severe outbreaks of stable flies have been associated with organic fertilizers, such as poultry litter and organic byproducts generated by sugar-alcohol mills. This is particularly evident in sugarcane fields, where fertigation with vinasse or fertilization with substrates like filter cake and vinasse sludge occur [98]. To prevent outbreaks linked to organic by-products from sugar and alcohol plants, it is essential to monitor the fly population and implement proper soil, straw, and vinasse management techniques [98].

Controlling horse flies (Tabanidae) is still challenging because of their high mobility and cryptic larval habitats. Insecticides, as noted by Foil and Hogsette [99], have limited effectiveness. However, visual traps such as the NZi trap developed by Mihok [100] have been successful in capturing biting flies and decreasing their impact on livestock.

When the disease is transmitted through the daily administration of oxytocin, it is important to interrupt its spread as soon as it is diagnosed. This can be achieved by suspending the use of oxytocin or by using disposable or previously disinfected needles for each animal. The use of oxytocin to induce milk letdown in zebu cattle is currently a widely used practice among dairy farmers using zebu breeds around the world. Some authors suggest that this practice increases milk production in zebu cows [101,102]. However, other authors have not found this to be the case and instead have shown that it can lead to reproductive issues [103], management problems, and animal welfare concerns. These issues are often evident through discomfort and reluctance to enter the milking parlor [22,104,105].

Considering that the main cause of the spread of trypanosomiasis in Brazil is the inappropriate use of oxytocin to induce milk let down in zebu cattle, some important procedures to eliminate oxytocin or use it without risks of disease transmission are outlined below [95,106].

(a) Heifers should be trained a few weeks before calving to familiarize themselves with the milking parlor. This involves simulating the udder preparation procedures, so that they become accustomed to the place and do not have difficulty releasing milk after calving.
(b) Before milking, the udder should be washed and the milk ejection reflex manually stimulated by massaging the teats.
(c) The application of exogenous oxytocin should be carefully planned and only be used in cows that truly have issues. This treatment is necessary when there is a risk of health problems in the mammary gland, caused by residual milk in the udder, which can lead to mastitis.
(d) A good milker should be able to identify animals that are difficult to milk and should massage the udder during mechanical milking and reposition the teat cups as needed. Therefore, oxytocin administration should only be performed in these animals.
(e) In Gir cows and their crossbreds, it is estimated that up to 35% of the cows in the herd may require oxytocin. Therefore, oxytocin administration should be carried out in these animals only.
(f) A long-term program should be established to select animals that can be milked without oxytocin.
(g) If oxytocin is administered, disposable needles and syringes should be used. An alternative is to use needles and syringes that have been disinfected after each administration. In addition, the drug should be administered in appropriate doses (<1 IU of oxytocin).
(h) It is necessary to define a medium- or long-term program to stop using oxytocin, since cows that are being administered oxytocin do not produce milk letdown when the product is not applied. Therefore, it may be necessary to wait until the next lactation to stop using oxytocin.

**Figure 4:**
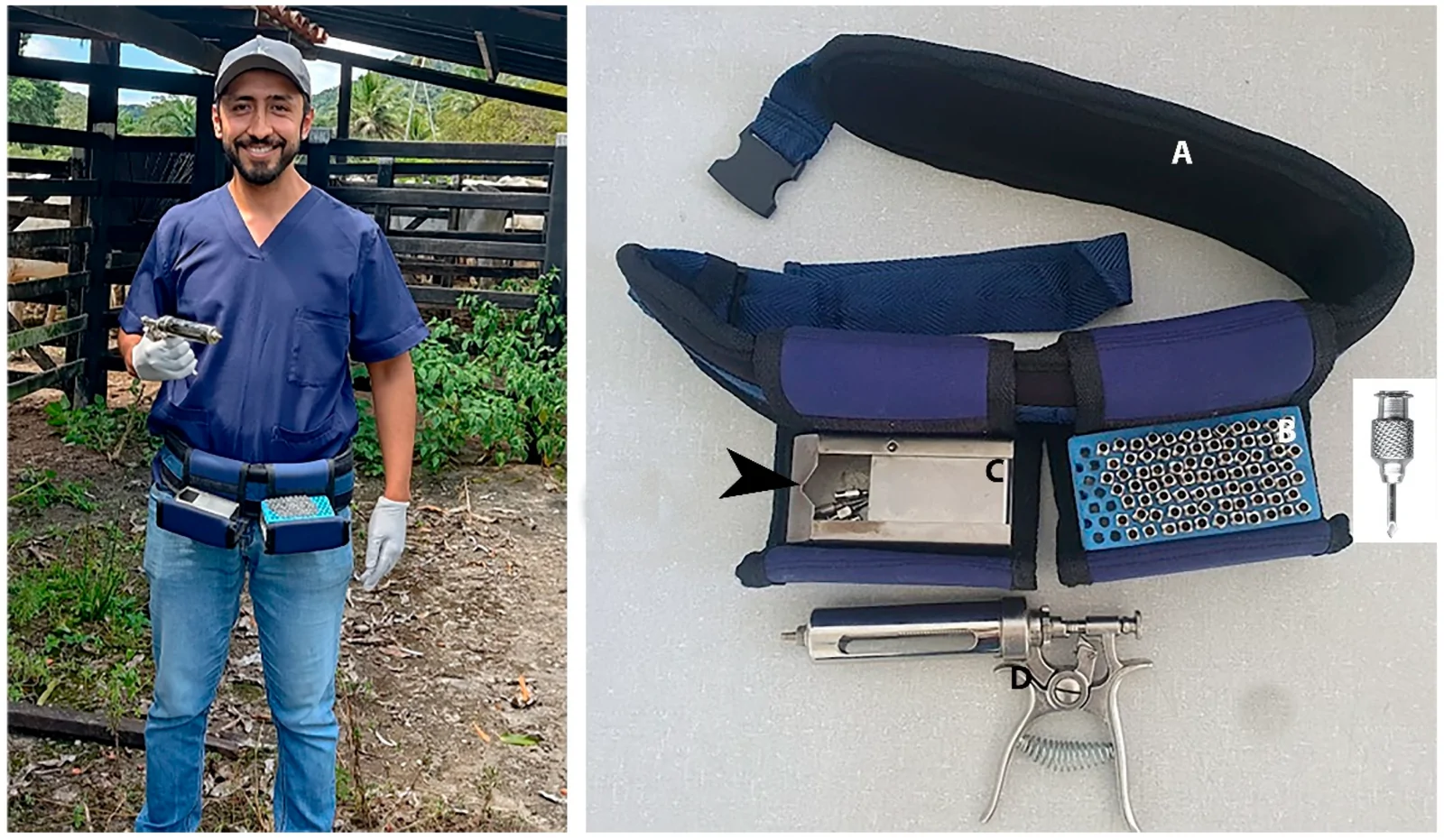
On the right, a device for single-use needles and their reuse after disinfection. Belt (A) with a needle holder with 91 disinfected needles ready for use (B) and a container (C) for the needles after use. This container has an angled edge (arrow) that aids in removing the needle from the syringe (D). On the left, the arrangement of the equipment on the person vaccinating the cattle.

It is also necessary to establish a system for administering medications and vaccines using disposable needles or needles that can be disinfected before reused. In Figure 4, we present an equipment that could serve as a model for using needles that can be disinfected after use on a single animal. Another approach could involve using two containers during vaccination: one for used needles to be disinfected and reused, and another for already disinfected needles that will be changed for each animal. It is important to note that the use of individual needles is not only necessary for the prophylaxis of trypanosomosis but also for other very important diseases such as anaplasmosis and, mainly, enzootic leukosis, which is one of the most common diseases in dairy cattle.

For the prophylaxis of trypanosomosis, it is crucial to exercise caution when acquiring animals, especially if they originate from areas with enzootic stability where the disease is present. Introducing animals into the herd should be timed when the population of Tabanidae and/or *S. calcitrans* is small. Additionally, it is imperative to refrain from sharing needles and syringes when administering injectable medications and vaccines. Currently, in Brazil, there is a need to raise awareness among dairy farmers who use oxytocin in zebu dairy cows about the need to eradicate or rationalize this practice, which is causing enormous losses to the country’s dairy industry.

## 7. Concluding Remarks

Trypanosomosis is an emerging disease in the southeastern region of Brazil and in some states in the Central–West and Northeastern regions. This disease is particularly prevalent in zebu dairy cattle due to the daily inoculation of oxytocin to induce milk letdown. Previously, sporadic cases of the disease were diagnosed in the Northeastern region, mainly in the semiarid where there is enzootic instability. Currently, most trypanosomiasis outbreaks occur in the Southeast and parts of the Northeast and Central–West regions, resulting from the use of daily oxytocin injections to induce milk production in dairy cattle. These outbreaks are also characterized by higher morbidity and mortality and faster spread than outbreaks transmitted by insects or by single injections of other drugs using the same syringe. In the previous century, trypanosomiasis was also reported in the Northern region and in the Pantanal of Mato Grosso. However, these regions of Brazil are now recognized as areas of enzootic stability, with abundant vectors year-round. In the South region, where the parasite is present but insect vectors are insufficient for transmission, no outbreaks have been recorded. In this region, due to the use of *BosTaurus* breeds, mainly Hosltein and Jersey, the use of oxytocin for milk letdown is completely discouraged.

The spread of trypanosomiasis occurs mainly through the transport of animals from areas of enzootic stability to free areas or vice versa.

Early diagnosis of the disease during outbreaks is crucial to implement effective control measures and prevent significant losses. Clinical signs, necropsy lesions, and epidemiological data must be observed, and parasitological or molecular techniques should be used to confirm the diagnosis for appropriate treatment and control measures.

To prevent the disease, dairy farmers should discontinue the routine use of oxytocin or limit its use to animals with milk release issues. Properly disinfecting needles and syringes or using disposable ones is also essential. In enzootic instability areas, precautions should be taken when introducing new cattle to the farms such as avoiding sharing needles during drug administration. Additionally, animals should not be introduced during the rainy season in regions with high Tabanidae populations.

Regions with an abundance of *S. calcitrans* such as those near sugar and ethanol plants, where vinasse is used as fertilizer, should be considered high risk areas for trypanosomosis. Similarly, areas with organic matter accumulation can lead to increased proliferation of *S. calcitrans*.

## Figures and Tables

**Table 1:** Epidemiologic data in outbreaks of trypanosomosis transmitted by hematophagous flies in cattle in Brazil.

State [Reference]	Breed	Production	Animal Introduction	Insects	Injectable Medication	Morbidity	Mortality
Pará, Amapá 6 farms ^a^ [16]	NI ^b^	NI	Yes	Tabanidae	NI	NI	NI
Mato Grosso [25]	NI	NI	NI	Tabanidae	NI	NI	NI
Tocantins [26]	Brahman	Beef cattle	Yes	Tabanidae	NI	9/250 (3.6%)	NI
Paraíba [1]	Brown Swiss	Milking cows Calves	Yes	Tabanidae	NI	64/130 (49%) 32/100 (32%)	11 (8.5%) 5/100 (5%)
Paraíba [27]	Brown Swiss	Milking cows	Yes	Tabanidae *Stomoxys calcitrans*	NI	17/36 (47%)	8/36 (22.2%)
Paraíba [27]	NI	Milking cows	Yes	Tabanidae *S. calcitrans*	NI	10/75 (13.3%)	7/75 (10.6%)
Paraíba [28] 3 outbreaks ^a^	Holstein x Brown Swiss	Calves	Yes	Tabanidae	NI	63–80%	15–20%
São Paulo [2]	Girolando Holstein	Milking cows and calves	Yes	*Haematobia irritans* *S. calcitrans*	NI	53/1080 (4.9%)	31/1080 (2.9%)
Pernambuco [29]	NI	Milking cows	No	Hematophagous flies	NI	22/80 (27.5%)	3/80 (3.75%)
Ceará [30]	Guzerá x Holstein	Milking cows	NI	NI	NI	48/210 (22.8%)	NI
Maranhão [31]	Girolando Holstein	Cows and calves	Yes	Tabanidae *S. calcitrans*	Yes	24/273 (8.79%)	6/273 (2.1%)

^a^ Five farms in Pará and one in Amapá. ^b^ Not informed.

**Table 2:** Epidemiologic data in 38 outbreaks of trypanosomosis transmitted by oxytocin inoculation in Brazil.

State [Reference]	Breed	Production	Animal Introduction	Morbidity	Mortality
São Paulo [32]	NI ^a^	Milking Cows	Yes	37/200 (18.5%)	15/200 (7.5%)
Pernambuco [11] Pernambuco [11]	Girolando NI	Milking cows Milking cows	NI NI	25/83 (25.3%) 25/75 (33.3%)	8/83 (9.6%) 20/75 (27.7%)
Sergipe [13]	NI	Milking Cows	Yes	3/15 (20%)	NI
Pernambuco [6] Pernambuco [6] Alagoas [6]	Girolando Girolando Girolando	Milking cows Milking cows Milking cows	Yes Yes Yes	NI NI NI	30/60 (50%) 8/62 (NI 15/102 (14.7%)
Goais ^b^ [7]	Girololando	Milking cows	Yes	51/161 (31%)	12/161 (22.7%)
Rio de Janeiro [9] 12 outbreaks	Girolando, Holstein	Milking cows Dry cows	Yes (in 9 of 12 farms)	10–90%	2.3–43.3%
Minas Gerais [23] 10 outbreaks	Girolando Holstein	Milking cows	NI	NI	0.55–41.7%
Espírito Santo [12]	Girolando	Milking cows	yes	10/22 (45.5%)	NI
Goiás [8] 24 outbreaks	Girolando, Gir, Holstein, Jersey Crossbreeds	Milking cows	Yes	8.84% ^c^	NI
Bahia [24]	Girolando	Milking cows	Yes	NI	10%
Bahia 2022 [21] 5 outbreaks	Girolando, Holstein, Gir	Milking cows	Yes	34/94 (35%)	0–18.9%
Minas Gerais [5]	Holstein	Milking cows	Yes	NI	6/37 (16.2%)
Bahia [10]	NI	Milking cows	Yes	NI	12/48 (25%)

^a^ Not informed. ^b^ The authors mention the occurrence of other 12 outbreaks in the same period in lactating cows; ^c^ The diseases occurred first in lactating cows and after in dry cows probably due to transmission by Tabanidae and *S. calcitrans*, which were abundant in the farms.

## Data Availability

No new data were created or analyzed in this study. Data sharing is not applicable to this article.
