# Correction: Riet-Correa et al. Trypanosomosis by *Trypanosoma* (*Dutonella*) *vivax* in Ruminants in Brazil: Epidemiology, Clinical Signs, Pathology, Diagnosis, and Control. *Vet. Sci*. 2025, *12*, 882

**DOI:** 10.3390/vetsci13101080

**Published:** 2026-10-13

**Authors:** Franklin Riet-Correa, Rodrigo Ferreira Krüger, Jose Augusto Bastos Afonso, João Alberto Negrão

**Affiliations:** 1 Postgraduate Program in Animal Science in the Tropics, Federal University of Bahia, Salvador 40170-110, Bahia, Brazil; 2 Ecology of Parasites and Vectors Group, Universidade Federal de Pelotas, Pelotas 96083-472, Rio Grande do Sul, Brazil; rodrigo.kruger@ufpel.edu.br; 3 Clínica de Bovinos, Universidade Federal Rural de Pernambuco—UFRPE, Garanhuns 52171-900, Pernambuco, Brazil; jose.basilva@ufrpe.br; 4 Basic Science Department, Faculty of Animal Science and Food Engineering (FZEA), University of São Paulo (USP), Av. Duque de Caxias Norte 225, Pirassununga 13635-900, São Paulo, Brazil; jnegrao@usp.br

In the original publication [1], the title mentioned the parasite as *Trypanosoma* (*Megatrypanum*) *vivax*. The correct form is *Trypanosoma* (*Dutonella*) *vivax*. Therefore, the title should be changed to “Trypanosomosis by *Trypanosoma* (*Dutonella*) *vivax* in Ruminants in Brazil: Epidemiology, Clinical Signs, Pathology, Diagnosis, and Control”. The authors state that the scientific conclusions are unaffected. This correction was approved by the Academic Editor. The original publication has also been updated.
